# Supplementary material for: Ca2+ Influx through TRPC Channels Is Regulated by Homocysteine–Copper Complexes
Source: Biomolecules. 2023 Jun 6;13(6):952. doi: 10.3390/biom13060952 (PMC10296383; doi:10.3390/biom13060952)
Supplement: Supplementary file 1 [file biomolecules-13-00952-s001.zip › biomolecules-2392403-supplementary.pdf]

# $Ca^{2+}$ influx through TRPC channels is regulated by homocysteine-copper complexes

**Gui-Lan Chen[1], Bo Zeng[1], Hongni Jiang[1], Nikoleta Daskoulidou[1], Rahul Saurabh[1], Rumbidzai J Chitando[1] and Shang-Zhong Xu[1,2*]**

1 Centre for Atherothrombosis and Metabolic Disease, Hull York Medical School, University of Hull, Hull, UK
2 Diabetes, Endocrinology and Metabolism, Hull York Medical School, University of Hull, Hull, UK
* Correspondence: sam.xu@hyms.ac.uk; Tel.: (44) 1482 465372

## Supplementary materials

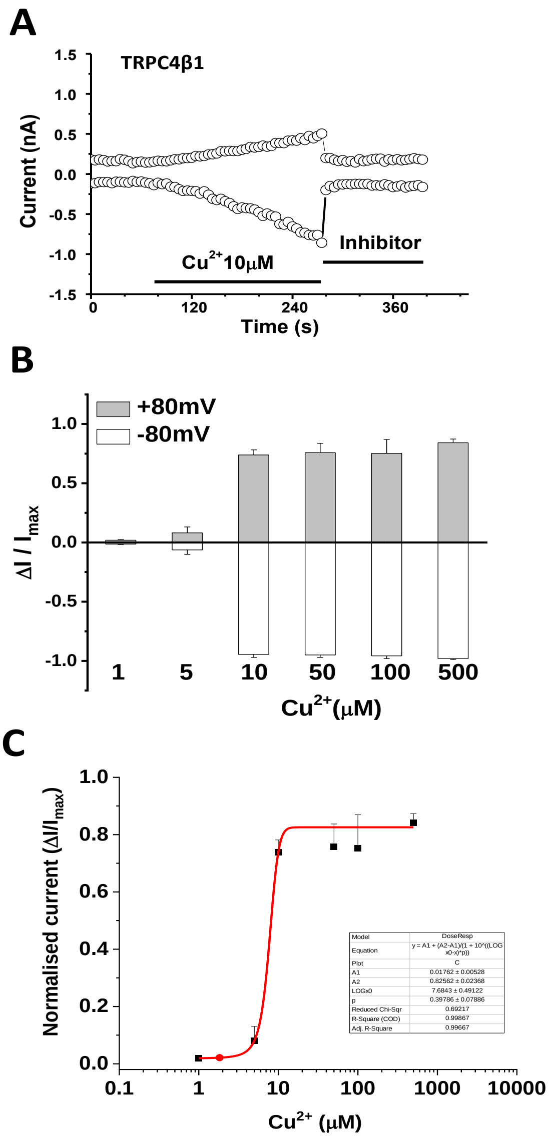


**Figure S1.** Activation of TRPC4β1 by $Cu^{2+}$ and the dose-response of $Cu^{2+}$ on TRPC4α. **A,** Example for the effect of $Cu^{2+}$ on TRPCβ1 current. **B,** Dose-dependent inhibitory effect on TRPC4α. **C,** The fitted dose-response curve showing an $EC_{50}$ of 7.1 μM measured at -80 mV (not shown) and 7.7 μM measured at +80 mV.

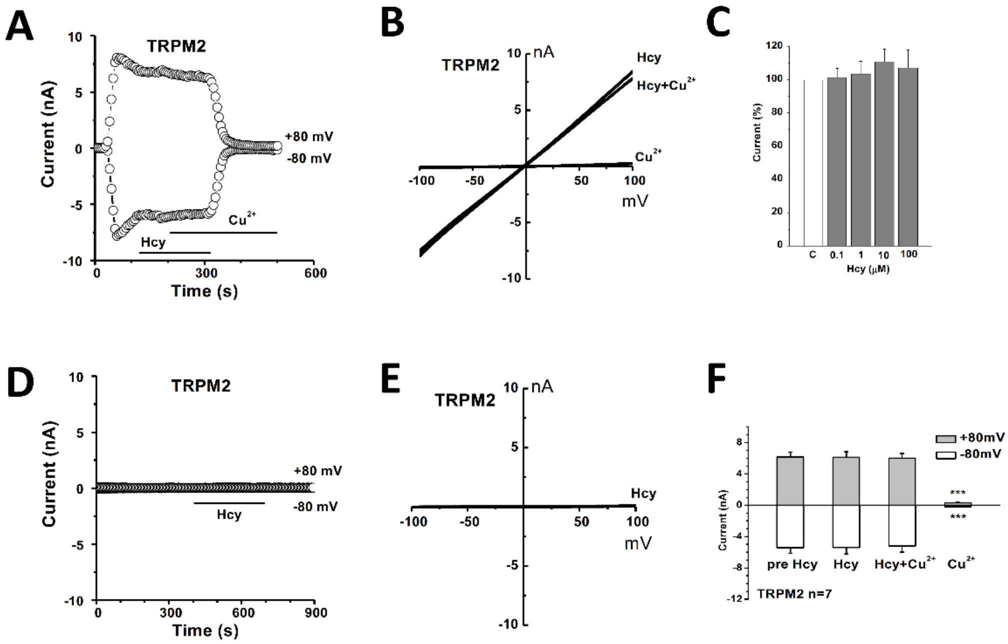


**Figure S2.** Effect of Hcy and copper on TRPM2 current. Whole cell current of TRPM2 was recorded using a pipette containing ADP-ribose. **A,** An example of time course of TRPM2 current. **B,** IV curves for (A). **C,** Mean data for the effect of Hcy and $Cu^{2+}$. **D-E**, Pipette without the activator ADP-ribose, the TRPM2 current was absent (no activation) and endogenous current in the non-induced cells was very small. **F,** Mean data for the effect on TRPM2 current. *** P<0.001.

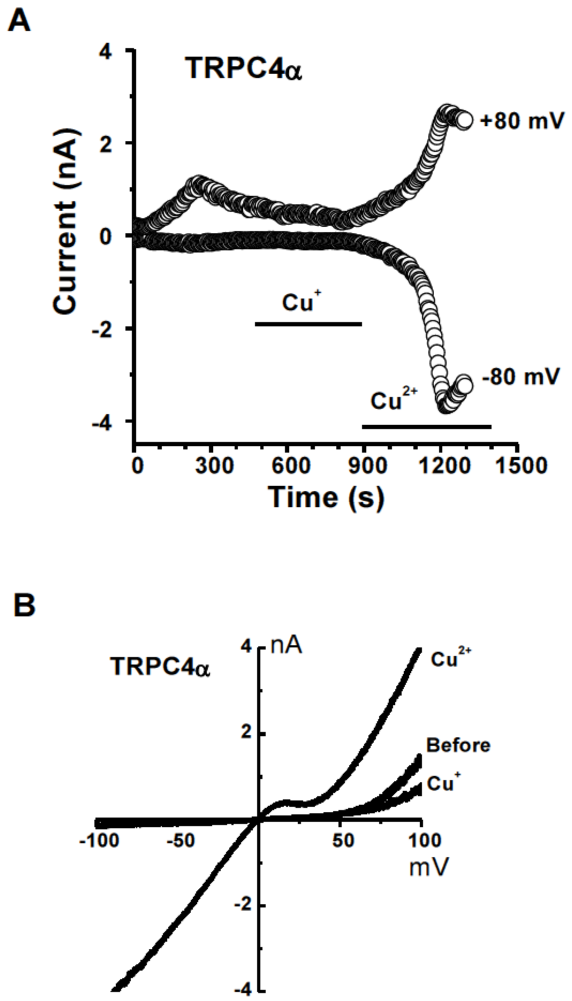


**Figure S3.** Example of monovalent copper (I) 1-butanethiolate on TRPC4α current. No effect was seen for monovalent $Cu^{+}$ 1-butanethiolate on TRPC4 current (n = 5). Copper (I) tetrakis(acetonitrile) copper(I) tetrafluoroborate) also has no effect on TRPC4α current (not shown). **A,** Time couse of TRPC4α current and the perfusion with 10 µM monovalent $Cu^{+}$ 1-butanethiolate followed by 10 µM $Cu^{2+}$. **B,** I-V curves for (A).

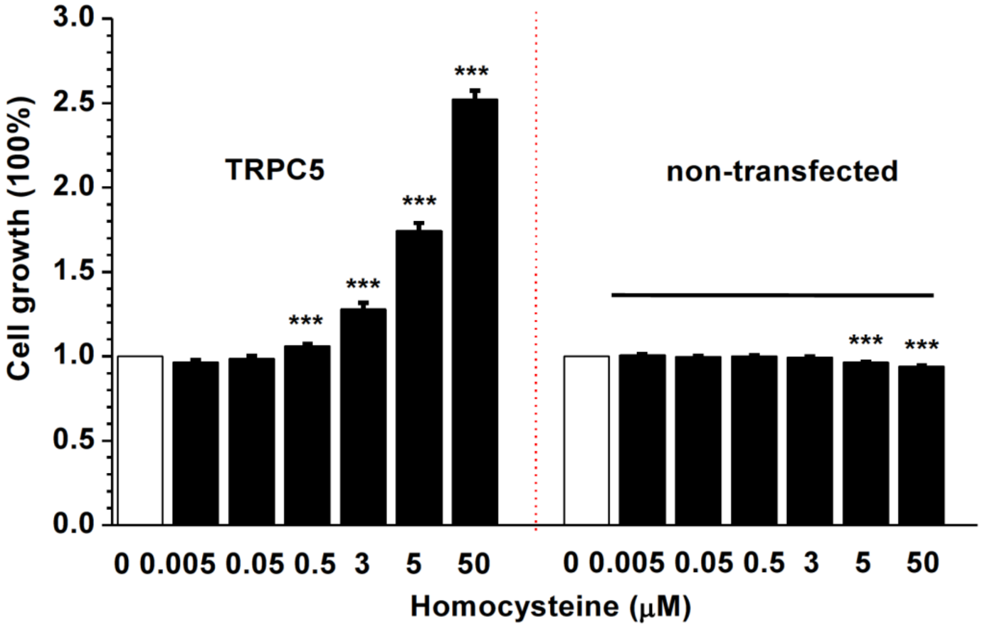


**Figure S4.** Hcy increased cell proliferation of T-Rex cells overexpressing TRPC5 (n=8 for each group). *** P<0.001.
